# Clonal expansion of chromosome-borne CTX-M-55 extended-spectrum β-lactamase-producing *Salmonella enterica* serovar Agona, Taiwan

**DOI:** 10.1128/spectrum.02979-24

**Published:** 2025-03-19

**Authors:** Ying-Shu Liao, Yu-Ping Hong, Bo-Han Chen, You-Wun Wang, Ru-Hsiou Teng, Yung-Chen Chien, Shiu-Yun Liang, Hsiao Lun Wei, Jui-Hsien Chang, Ming-Hao Yang, Chi-Sen Tsao, Chien-Shun Chiou

**Affiliations:** 1 Center for Diagnostics and Vaccine Development, Centers for Disease Control, Ministry of Health and Welfare, Taichung, Taiwan; 2 Taipei City Hospital Renai Branch 156947, Taipei City, Taiwan; Rush University Medical Center, Chicago, Illinois, USA

**Keywords:** *Salmonella*, antimicrobial resistance, multidrug-resistant, resistance genomic island, molecular epidemiology

## Abstract

We investigated emerging multidrug-resistant (MDR) *Salmonella enterica* serovar Agona in Taiwan. These MDR strains commonly harbored the efflux pump activator gene *ramAp* and up to 15 resistance genes, including *aac (3)-IId*, *aadA22*, *aph(3')-Ia*, *aph (6)-Id*, *arr-2*, *bla*_CTX-M-55_, *bla*_LAP-2_, *bla*_TEM-1_, *dfrA14*, *floR*, *lnu(F*), *qnrS13*, *sul2*, *sul3*, and *tet(A*), within IncHI2-IncHI2A plasmids or diverse chromosomal resistance genomic islands (RGIs). These newly emerging MDR strains comprised 44.9% and 74.4% of *S*. Agona isolates collected in 2023 and 2024, respectively. Through analysis of chromosomal insertion sites, we identified 11 distinct RGIs. Strains containing RGI_SA11 became predominant, comprising 59.2% of *S*. Agona isolates collected in 2024.

**IMPORTANCE:**

The emergence and clonal expansion of MDR *S*. Agona represent a growing public health concern. This study identifies a significant shift in resistance mechanisms, driven by chromosomally integrated resistance genomic islands (RGIs), which likely originated from IncHI2–IncHI2A plasmids. The chromosomal integration of antimicrobial resistance genes enhances the evolutionary fitness of these strains, facilitating their persistence and dissemination in both human and animal populations. These findings underscore the urgent need for enhanced surveillance, targeted interventions, and global antimicrobial stewardship to curb the spread of MDR pathogens and safeguard public health.

## INTRODUCTION

*Salmonella enterica* serovar Agona (*S*. Agona) is commonly isolated from humans and food animals, including poultry and livestock (1). In Taiwan, *S*. Agona ranked as the 6th most prevalent serovar in human salmonellosis cases from 2004 to 2022 and was frequently identified in chicken meat and pork carcasses (2–4). *S*. Agona has not historically been classified among the most antimicrobial-resistant serovars in Taiwan (3).

In February 2019, we received two extensively drug-resistant (XDR) *Salmonella* isolates from a single patient, collected five days apart at a hospital. These two isolates were resistant to ampicillin, chloramphenicol, trimethoprim-sulfamethoxazole, fluoroquinolones, and third-generation cephalosporins, aligning with the definition of XDR *S*. Typhi (5). Genomic analysis revealed that the two XDR isolates were *S*. Agona (R19.0144) and *S*. Goldcoast (R19.0145), carrying highly similar IncHI2–IncHI2A plasmids. The plasmids from the two isolates harbored an efflux pump activator gene *ramAp* (6) and 15 antimicrobial resistance genes (ARGs), *aac (3)-IId*, *aadA22*, *aph(3')-Ia*, *aph (6)-Id*, *arr-2*, *bla*_CTX-M-55_, *bla*_LAP-2_, *bla*_TEM-1_, *dfrA14*, *floR*, *lnu(F*), *qnrS13*, *sul2*, *sul3*, and *tet(A*), which had previously been identified in plasmid pR18.0877_278 k from *S*. Goldcoast strain R18.0877 (7). This plasmid-borne *ramAp* confers elevated resistance to multiple antimicrobials, including azithromycin, chloramphenicol, ciprofloxacin, nalidixic acid, sulfamethoxazole, trimethoprim, tetracycline, and tigecycline, by enhancing efflux pump activity (6).

In 2021, we further analyzed two XDR *S*. Agona isolates recovered from another patient, one of which developed resistance to ertapenem (a carbapenem) during therapy. Both isolates harbored a defective *ompC* gene, caused by the insertion of a 160 kb IS15DI composite transposon. This transposon carried *ramAp* and 12 ARGs identical to those found in pR18.0877_278 k (8). Since 2023, we have observed a rising trend in multidrug-resistant (MDR) *S*. Agona, defined as resistance to at least three distinct antimicrobial classes. In this study, we investigate the epidemiological trends, antimicrobial resistance, and genomic characteristics of *S*. Agona isolates, with a particular focus on those collected between 2021 and 2024.

## MATERIALS AND METHODS

### *Salmonella* isolates

*Salmonella* isolates obtained from human cases of salmonellosis were collected from collaborating hospitals across Taiwan from 2021 to 2024, as part of a disease surveillance initiative (PulseNet Taiwan), which has been operated since 2004. This study was approved by the Institutional Review Board of the Taiwan Centers for Disease Control (Taiwan CDC), which granted a waiver for informed consent. All bacterial isolates were re-confirmed as *Salmonella* using the MALDI Biotyper (Bruker Corp., USA). The isolates were subsequently genotyped following the standardized PulseNet pulsed-field gel electrophoresis (PFGE) protocol (9). Serotypes were determined by comparing PFGE patterns with those in the *Salmonella* PFGE database established by Taiwan CDC (10). Additionally, five ESC-resistant, MDR *S*. Agona isolates collected from human and animal sources before 2021 were included in the phylogenetic analysis and resistance mechanism assessment.

### Antimicrobial susceptibility testing (AST)

All *S*. Agona isolates collected from humans between 2021 and 2024 were tested to determine the minimum inhibitory concentration (MIC). AST was performed using the micro-broth dilution method with Sensititre EUVSEC3 panels (TREK Diagnostic Systems LTD., West Sussex, UK). The panel included 14 antimicrobials: azithromycin, ampicillin, cefotaxime, ceftazidime, meropenem, nalidixic acid, ciprofloxacin, gentamicin, chloramphenicol, sulfamethoxazole, trimethoprim, tetracycline, colistin, and tigecycline. The testing procedure followed the manufacturer’s instructions. Except for colistin, tigecycline, and azithromycin, MIC results were interpreted using the guidelines established by the Clinical and Laboratory Standards Institute (CLSI) (11). The MIC results for colistin and tigecycline were interpreted using the standards set by the European Committee on Antimicrobial Susceptibility Testing (EUCAST) (12). For azithromycin, resistance was defined as MIC ≧ 32 mg/L and susceptibility as MIC ≦ 16 mg/L, according to the interpretive criteria set by CDC NARMS (https://www.cdc.gov/narms/about/antibiotics-tested.html). Additionally, ciprofloxacin nonsusceptibility (NS) encompasses isolates classified as either resistant or intermediate according to susceptibility testing. MDR is defined as resistance to at least three antimicrobial classes, including macrolides (azithromycin), β-lactams (ampicillin, cefotaxime, ceftazidime, or meropenem), quinolones (nalidixic acid or ciprofloxacin), aminoglycosides (gentamicin), phenicols (chloramphenicol), folate pathway inhibitors (trimethoprim and sulfamethoxazole), tetracyclines (tetracycline), polymyxins (colistin), and glycylcyclines (tigecycline).

### Whole genome sequencing (WGS)

Seventy-two MDR S. Agona isolates, exhibiting resistance to ESCs, were recovered from humans, pigs, ducks, chickens, and chicken meat between 2015 and 2024 and selected for WGS to identify resistance determinants and their associated genetic vehicles. WGS was conducted using both the Illumina MiSeq platform (Illumina Inc., California, USA) and/or the Oxford Nanopore Technologies (ONT) platform (Oxford Nanopore Technologies Limited, Oxford, UK). Illumina sequence reads were assembled using SPAdes version 3.15.3, while ONT sequencing utilized Dorado v0.5.0 for base calling with the dna_r10.4.1_e8.2_400bps_sup@4.3.0 model and Flye v2.9.2 for chromosome assembly. Plasmid assembly was performed using Plassembler v1.6.0 (13), and sequence polishing was completed with Medaka v1.11.3 using the r1041_e82_400bps_sup_v4.3.0 model (https://github.com/nanoporetech/medaka). For isolates sequenced with both Illumina and ONT platforms, nanopore assemblies were polished using Illumina reads with Polypolish v0.6.0 (14) and Pypolca v0.3.0 (15, 16) to improve genome sequence accuracy.

### Polymerase chain reaction (PCR)

PCR was employed to detect *ramAp*, group 1 of *bla*_CTX-M_ (including *bla*_CTX-M-55_), IncHI2 and IncHI2A replicons, and the insertion of *S*. Agona resistance genomic island_11 (RGI_SA11). The primers used are listed in Table S1.

### Analysis of WGS data

We used the AMRFinderPlus pipeline provided in the NCBI database, as well as ResFinder and PlasmidFinder supplied by the Center for Genomic Epidemiology (http://www.genomicepidemiology.org), to determine antimicrobial resistance genes, resistance-relevant mutations, conventional sequence types (STs), and plasmid incompatibility (Inc) types from the assembled contigs of each isolate.

### Construction of PFGE dendrogram

The genetic relationships among *S*. Agona isolates were assessed using clustering analysis of their PFGE patterns, utilizing the tools provided by BioNumerics version 6.6 (Applied Maths). The similarity between two PFGE patterns was measured using the Dice similarity coefficient. The resulting dendrogram in Fig. S1 was generated using the unweighted pair group method with the arithmetic mean (UPGMA) algorithm, with a setting of 1.5% optimization and 0.75% tolerance.

### Comparison of genetic maps of plasmids

We compared the genetic maps of pR18.0246_278 k (from *S*. Agona R18.0246), pR18.0877_278 k (from *S*. Goldcoast R18.0877), pR19.0144_302 k (from *S*. Agona R19.0144), and pR19.0145_278 k (from *S*. Goldcoast R19.0145). The maps were generated using the Easyfig software (https://mjsull.github.io/Easyfig/). In Fig. S2, antimicrobial resistance genes and *ramAp* are highlighted in red, while insertion sequences IS26 or its variant IS15DI are marked in green.

### Genetic relatedness among IncHI2–IncHI2A plasmids

**Fig 1:**
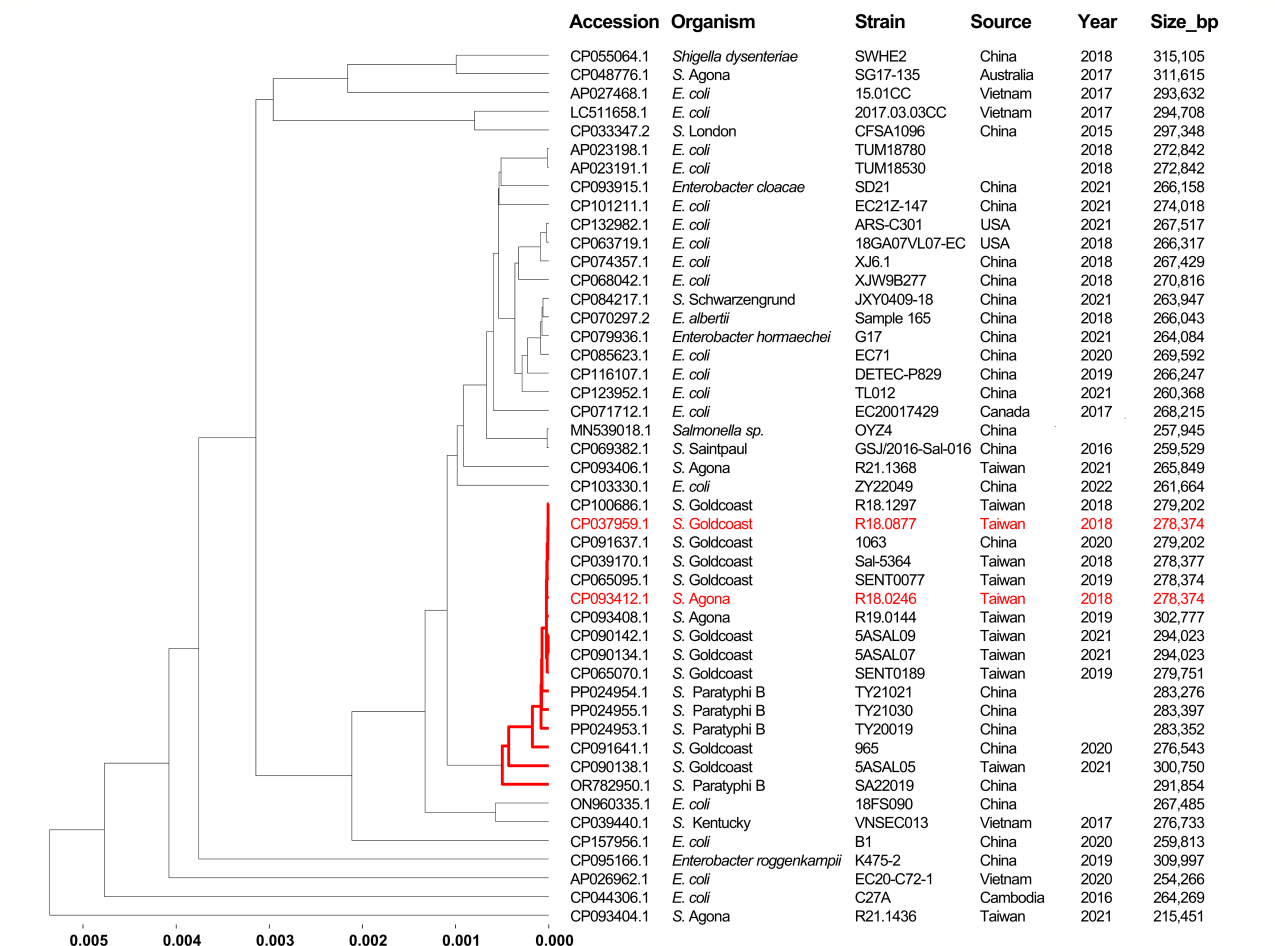
Phylogenetic tree of closely related IncHI2–IncHI2A plasmids identified in this study and retrieved from the NCBI database. All plasmids carry *ramAp* and *bla*_CTX-M-55_. The tree was constructed using the single-linkage clustering algorithm. Plasmids from *S*. Goldcoast R18.0877 and *S*. Agona R18.046, which are nearly identical, are highlighted in red. The cluster containing plasmids with Mash distances of less than 0.001 (corresponding to less than 0.1% genetic difference) is indicated with red lines.

To investigate the distribution of IncHI2–IncHI2A plasmids related to pR18.0246_278 k from *S*. Agona R18.0246, the nucleotide sequence of *ramAp* was used to query the NCBI nucleotide database via the BLAST tool. Entries containing *ramAp* sequences were retrieved, and plasmids were selected for further analysis of their incompatibility types and associated antimicrobial resistance genes. Plasmids carrying both IncHI2–IncHI2A replicons and *bla*_CTX-M-55_ were included in the phylogenetic analysis. Pairwise distances between plasmids were calculated using Mash (17), and a phylogenetic tree was constructed using the single-linkage algorithm. Plasmids with a pairwise distance of <0.006 from pR18.0246_278 k were included in Fig. 1, demonstrating close genetic relatedness among these plasmids. Details of these plasmids, including their genetic features and associated resistance genes, are summarized in Table S2.

### Phylogenetic analysis of ESC-resistant *S*. Agona isolates

The phylogenetic analysis of 72 ESC-resistant *S*. Agona isolates was conducted using core genome single nucleotide polymorphism (cgSNP) profiles. The cgSNP profiles were generated with ska.rust v0.3.7, using the reference-free approach (18). Phylogenetic inference was performed using the maximum likelihood method implemented in IQ-TREE v2.2.2 (19) and the resulting phylogenetic tree was visualized with iTOL (20).

### Comparison of RGI_SAs and RGI_SA11 variants

**Fig 2:**
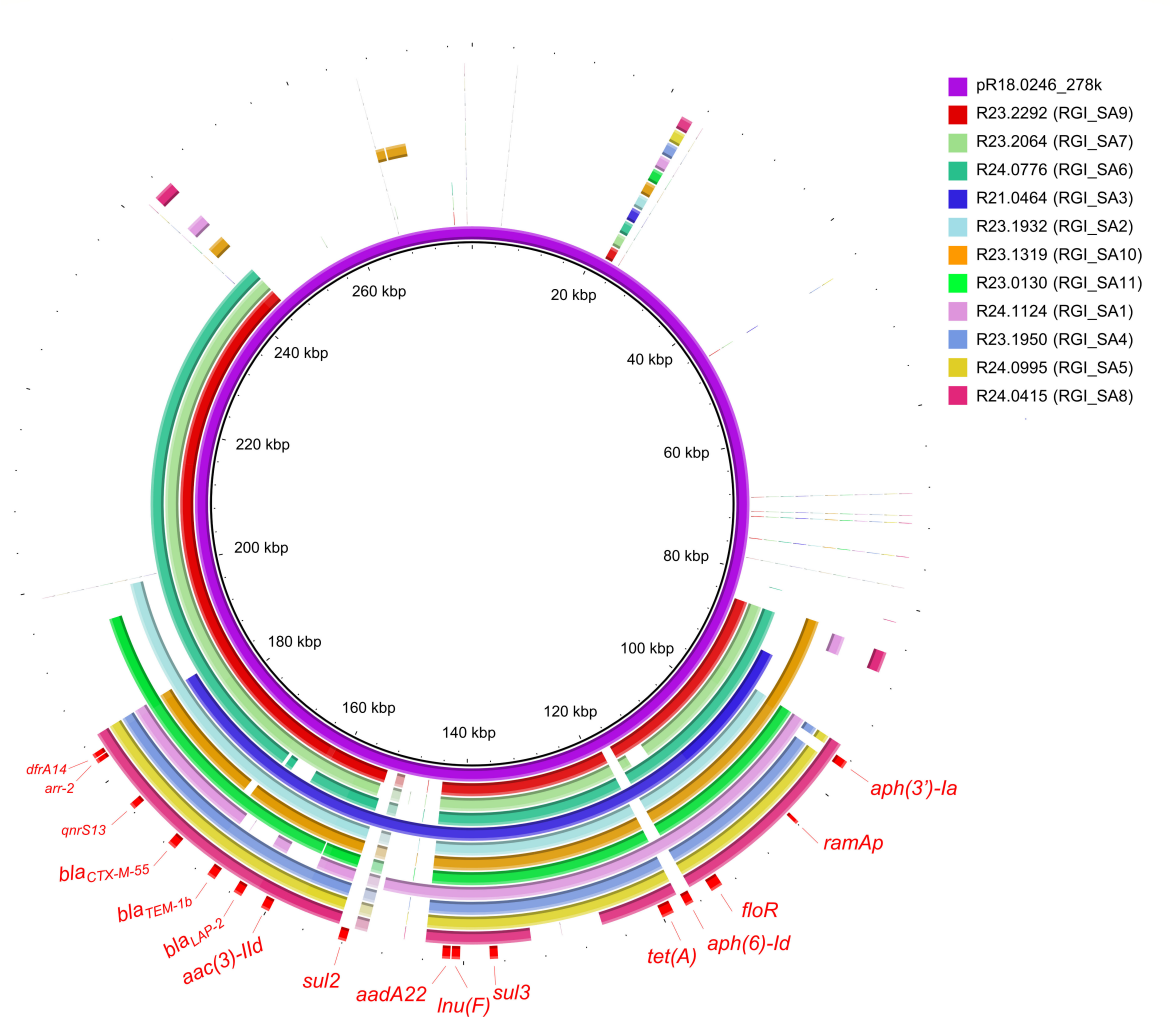
Genetic maps of 11 *S*. Agona resistance genomic islands (RGI_SAs). Plasmid pR18.0246_278 k was used as the reference for comparative analysis.

The sequence of pR18.0246_278 k from *S*. Agona R18.0246 was used as the reference for comparing 11 *S*. Agona resistance genomic islands (RGI_SAs) (Fig. 2) and 8 RGI_SA11 variants (Fig. S3). The comparison was performed using the BLAST Ring Image Generator (BRIG) pipeline (http://brig.sourceforge.net/) (21).

## RESULTS AND DISCUSSION

Our surveillance data revealed that *S*. Agona was the third most prevalent serovar, responsible for 6.2% (360/5,783) of human salmonellosis cases between 2021 and 2024, following *S*. Enteritidis (42.2%) and *S*. Typhimurium (21.1%). Notably, the isolation rate of *S*. Agona increased to 10.7% in 2024 (Table 1). Antimicrobial susceptibility testing indicated high resistance rates among the isolates collected in 2023 and 2024. These isolates exhibited high rates (22.8%–48.6%) of resistance to ampicillin, ESCs (e.g., cefotaxime and ceftazidime), nalidixic acid, ciprofloxacin, gentamicin, chloramphenicol, trimethoprim, sulfamethoxazole, tetracycline, and tigecycline (Table 1). Moreover, the proportion of MDR *S*. Agona isolates, defined as resistance to at least three antimicrobial classes, increased significantly from 11.8% in 2022 to 72.0% in 2024, with an overall prevalence of 40.8%. Clustering analysis of pulsed-field gel electrophoresis patterns demonstrated that MDR *S*. Agona isolates, with resistance to ESCs, were grouped into five distinct clusters, with a few isolates dispersed across the dendrogram, suggesting notable genetic diversity among these strains (see Fig. S1 in the supplemental material).

**TABLE 1:** Distribution of *S*. Agona isolates and their antimicrobial resistance profiles

	2021	2022	2023	2024	Total
All *Salmonella* serovars	1,486	1,490	1,643	1,164	5,783
*S*. Agona (%)	98 (6.6)	68 (4.6)	69 (4.2)	125 (10.7)	360 (6.2)
Resistance in *S*. Agona (%)					
Azithromycin	15.3	0	10.1	3.2	7.2
Ampicillin	19.4	16.2	46.4	68.8	41.1
Cefotaxime	18.4	16.2	36.2	58.4	35.3
Ceftazidime	20.4	16.2	37.7	57.6	35.8
Meropenem	0	0	0	0	0
Nalidixic acid	16.3	8.8	31.9	30.4	22.8
Ciprofloxacin	15.3	11.8	33.3	40.8	26.9
Ciprofloxacin^NS^	16.3	11.8	44.9	55.2	34.4
Gentamicin	13.3	11.8	40.6	49.6	30.8
Chloramphenicol	22.4	11.8	40.6	65.6	38.9
Trimethoprim	15.3	10.3	46.4	72.0	40.0
Sulfamethoxazole	45.9	11.8	47.8	71.2	48.6
Tetracycline	17.3	11.8	49.3	66.4	39.4
Colistin	0	0	0	0.8	0.3
Tigecycline	16.3	13.2	34.8	48.8	30.6
MDR^a^	17.3	11.8	46.4	72.0	40.8

^a^ MDR: Defined as resistance to at least three antimicrobial classes, including macrolides (azithromycin), β-lactams (ampicillin, cefotaxime, ceftazidime, or meropenem), quinolones (nalidixic acid or ciprofloxacin), aminoglycosides (gentamicin), phenicols (chloramphenicol), folate pathway inhibitors (trimethoprim-sulfamethoxazole), tetracyclines (tetracycline), polymyxins (colistin), and glycylcyclines (tigecycline). Ciprofloxacin^NS^: ciprofloxacin nonsusceptibility includes both resistant and intermediate categories.

To investigate their ARGs, associated genetic vehicles, and phylogenetic relationships, we performed whole-genome sequencing (WGS) on 72 MDR S. Agona isolates with resistance to ESCs. The ESC-resistant isolates were recovered from humans, pigs, ducks, chickens, and chicken meat between 2015 and 2024. Genomic analysis revealed that all *S*. Agona isolates belonged to ST13 and carried *fosA7.2* within their chromosomes. Among these, 66 of 72 isolates carried *ramAp*, and 70 harbored *bla*_CTX-M-55_ (Table S3). Two isolates lacked both ramAp and *bla*_CTX-M-55_. In addition to fosA7.2, the *bla*_CTX-M-55_-carrying isolates harbored up to 15 ARGs, including *aac (3)-IId*, *aadA22*, *aph(3')-Ia*, *aph (6)-Id*, *arr-2*, *bla*_CTX-M-55_, *bla*_LAP-2_, *bla*_TEM-1_, *dfrA14*, *floR*, *lnu(F*), *qnrS13*, *sul2*, *sul3*, and *tet(A*), located either within IncHI2–IncHI2A plasmids or chromosomal regions.

Among the 70 *bla*_CTX-M-55_-carrying isolates, 24 harbored ARGs within IncHI2–IncHI2A plasmids. The IncHI2–IncHI2A plasmids carrying 15 ARGs were first identified in 2018 from a patient’s *S*. Agona isolate (R18.0246). Similar plasmids were subsequently identified in isolates from pigs, ducks, chickens, and chicken meat (Table S3). The plasmid from *S*. Agona R18.0246, designated pR18.0246_278 k (accession number CP093412.1), shared a nearly identical sequence with plasmids from *S*. Goldcoast isolates R18.0877 and R19.0145, differing by only 7 and 9 bp, respectively (Fig. S2). In a traceback study, we searched the NCBI database for IncHI2–IncHI2A plasmids carrying *ramAp* and *bla*_CTX-M-55_ to assess their genetic relatedness. We identified closely related IncHI2–IncHI2A plasmids from various *Salmonella* serovars including Agona, Goldcoast, Kentucky, London, Paratyphi B, and Saintpaul, as well as from several *Enterobacteriaceae* species, including *Shigella dysenteriae*, *Escherichia coli*, *E. albertii*, *Enterobacter cloacae*, *Enterobacter hormaechei*, and *Enterobacter roggenkampii* (Fig. 1). These plasmids carried varying numbers of the aforementioned 15 ARGs, with some harboring additional ARGs. The plasmid from *S*. Agona R18.0246 displayed close genetic similarity to those found in *S*. Goldcoast and *S*. Paratyphi B isolates, originating from Taiwan and China.

Of the 70 *bla*_CTX-M-55_-carrying isolates, 46 harbored the IncHI2–IncHI2A plasmid-associated ARGs within their chromosomes. These *S*. Agona resistance genomic islands, referred to here as RGI_SAs, were identified as composite transposons flanked by IS26 or its variant IS15DI. Through the analysis of the chromosomal insertion sites, we identified 11 RGI_SAs (Table 2). However, we were not able to determine the insertion sites for five isolates, possibly due to further reassortment of the genomic islands. Sequence comparisons suggested that these RGI_SAs were most likely originated from IncHI2–IncHI2A plasmids (Fig. 2). The most prevalent genomic island, RGI_SA11, was integrated into a gene encoding a LysR family transcriptional regulator (Table 2). Another island, RGI_SA6, inserted within the *ompC*, was identified in six isolates originating from humans and a duck (Table S3).

**TABLE 2:** Characteristics of *S*. Agona resistance genomic islands (RGI_SAs)

RGI	No. Isolates^*a*^	Reference strain	Size, bp	Insertion site^*b*^	Location of RGI	Flanked by	Direct repeat
RGI_SA1	1	R24.1124	85,098	152,808‒152,815	MFS transporter	IS15DI	CTCGCCAC
RGI_SA2	2	R23.1932	91,603	1,124,475‒1,124,482	Carboxymuconolactone decarboxylase family protein	IS15DI	CTTTACTG
RGI_SA3	1	R21.0464	94,940	1,325,152‒1,325,159	*cadB*	IS15DI	CCGGGAAG
RGI_SA4	1	R23.1950	75,470	1,453,538‒1,460,144	*etuA*	IS15DI	Deleted
RGI_SA5	5	R24.0995	74,642	1,460,145‒1,460,152	DUF1963 domain-containing protein	IS15DI	CAATTTCC
RGI_SA6	6	R24.0776	140,641	1,666,381‒1,666,388	*ompC*	IS15DI	CATGAAAG
RGI_SA7	1	R23.2064	144,474	1,908,495‒1,908,502	*pduC*	IS15DI	AGCTCGGT
RGI_SA8	2	R24.0415	70,280	2,920,108‒2,920,115	*hpaA*	IS26	GAAAAAAC
RGI_SA9	9	R23.2292	149,020	3,186,374‒3,186,381	ABC transporter ATP-binding protein	IS15DI	GCCGTTCG
RGI_SA10	2	R23.1319	90,475	3,611,493‒3,611,500	Between the Ail/Lom family outer membrane beta-barrel protein and a hypothetical protein	IS26	CAATTTCG
RGI_SA11	18	R23.0130	88,074	4,016,284‒4,016,291	LysR family transcriptional regulator	IS15DI	AAGCGAAT

^*a*^ The number of isolates with the RGI_SA and its variants among the 72 ESC-resistant isolates (Table S3).

^*b*^ Refer to the genomic sequence of *S*. Agona strain R18.0246 (accession no. CP093411.1).

**Fig 3:**
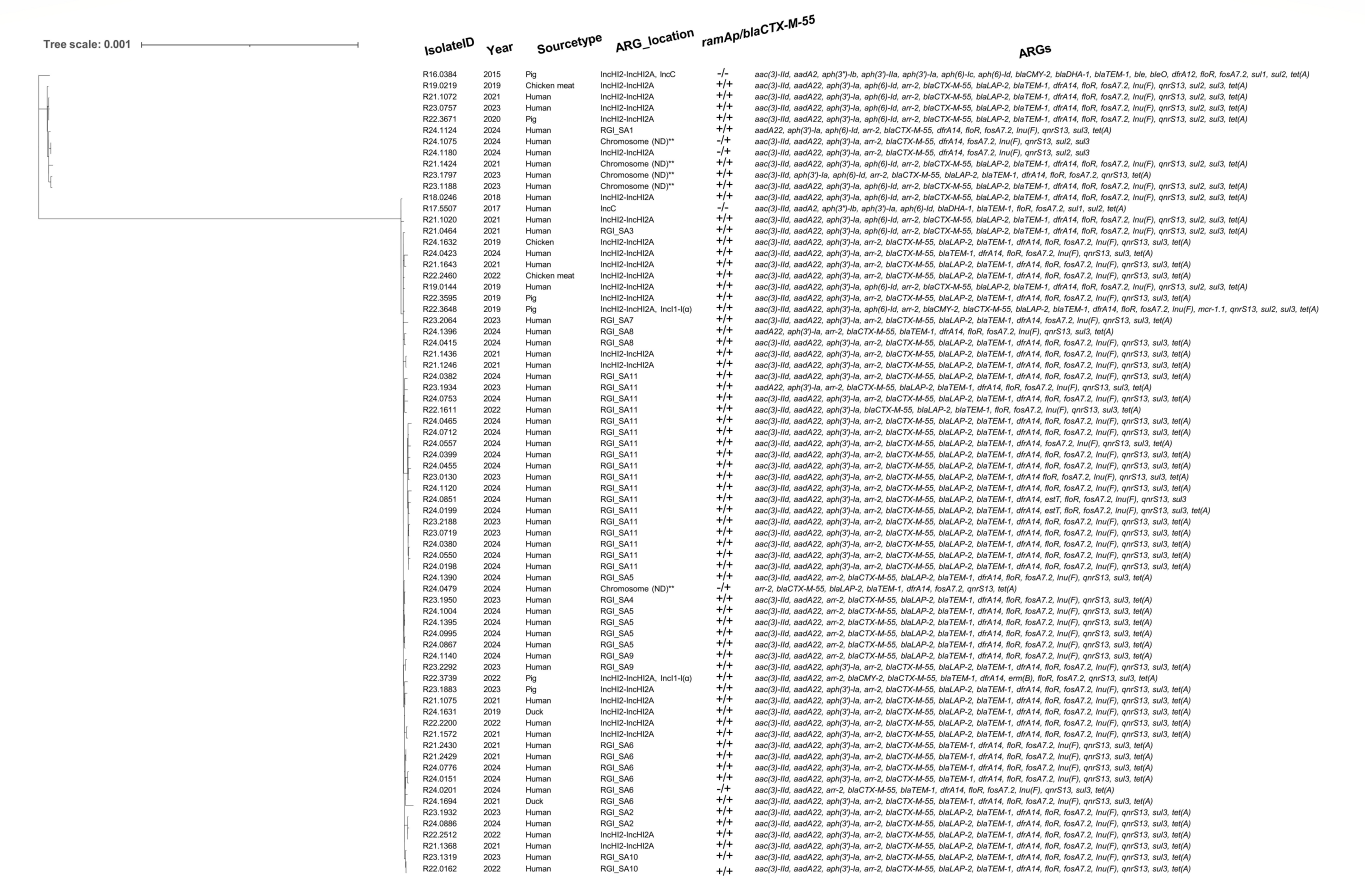
Phylogenetic tree for 72 ESC-resistant *S*. Agona isolates. The tree was constructed with cgSNP profiles using the maximum likelihood algorithm. RGI_SA refers to the *S*. Agona resistance genomic island.

Phylogenetic analysis revealed that the 72 ESC-resistant isolates clustered into two distinct genetic lineages (Fig. 3). Isolates carrying IncHI2–IncHI2A plasmids exhibited considerable diversity, indicating that plasmid-borne ARGs may have undergone frequent horizontal transfer between *Salmonella* strains via plasmid conjugation. In contrast, isolates sharing the same RGI type displayed a closer genetic relationship, suggesting clonal expansion following the acquisition of the RGI. Notably, RGI_SA11 had diversified into multiple variants, varying in sequence length and the number of ARGs they carry (Fig. S3).

**Fig 4:**
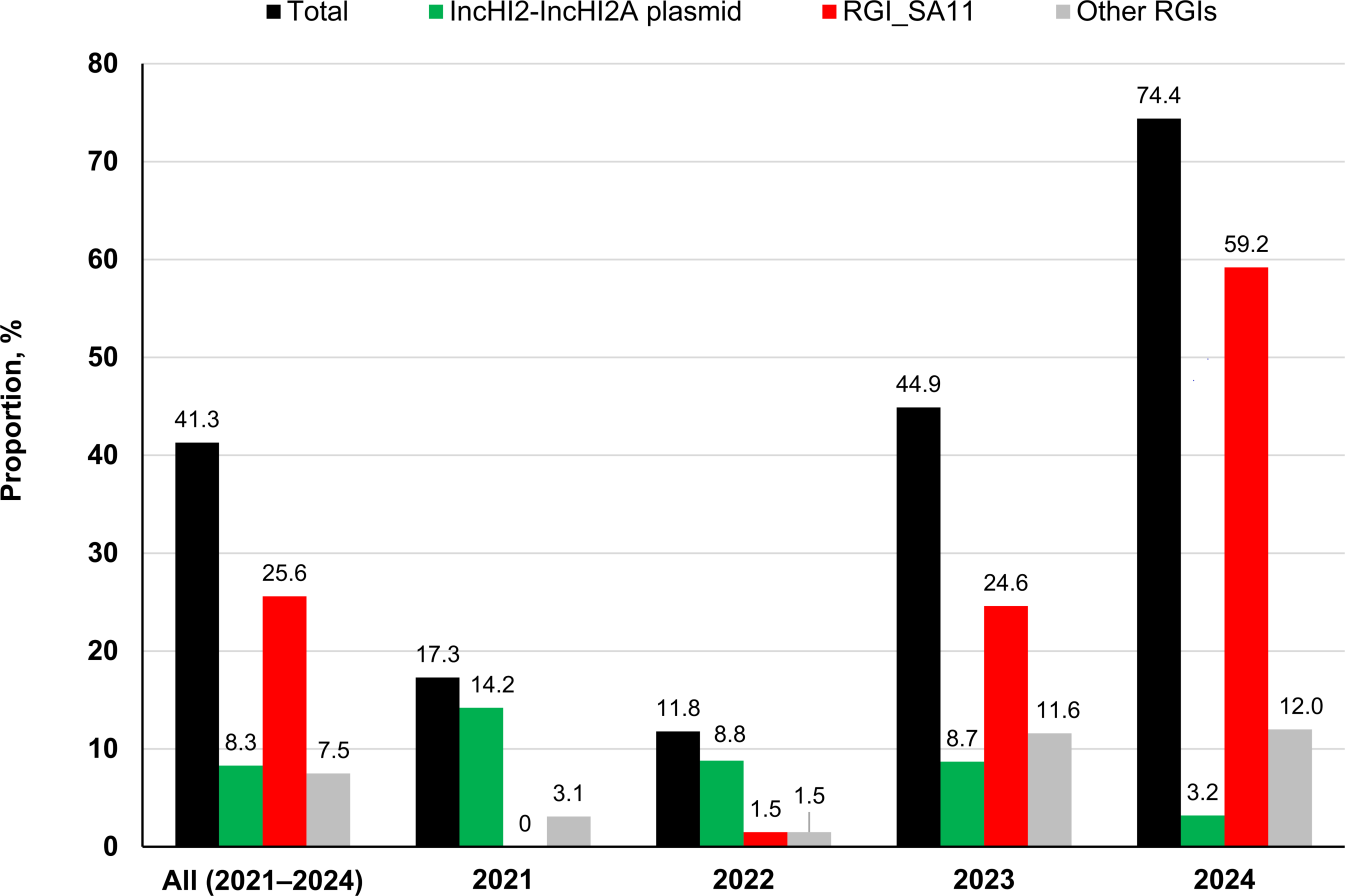
Prevalence of MDR *S*. Agona strains and the associated mobile genetic elements carrying antimicrobial resistance genes. The "total" refers to the combined proportion of isolates carrying the following elements: IncHI2–IncHI2A plasmids, RGI_SA11, and other *S*. Agona RGIs. RGI refers to resistance genomic island.

We utilized PCR to detect the presence of *ramAp*, *bla*_CTX-M-55_, IncHI2–IncHI2A replicons, and the insertion of RGI_SA11 in isolates lacking WGS data to assess the prevalence of this newly emerging MDR *S*. Agona between 2021 and 2024. The combined results of WGS and PCR analysis revealed that 41.3% (149/360) of the *S*. Agona isolates were classified as newly emerging MDR strains, with a marked rise in prevalence observed in 2023 and 2024 (Fig. 4). Notably, these MDR strains comprised 74.4% of *S*. Agona isolates collected in 2024. Among the 360 isolates, 30 (8.3%) carried ARGs on IncHI2–IncHI2A plasmids, with prevalence decreasing from 14.2% in 2021 to 3.2% in 2024. Additionally, 119 isolates (33.1%) harbored ARGs in their chromosomes. Of these, 92 isolates (25.6%) contained ARGs within RGI_SA11, while 27 (7.5%) carried ARGs in other RGIs. RGI_SA11 was initially identified in a single isolate in 2022 but became predominant by 2023 and 2024, representing 59.2% of *S*. Agona isolates collected in 2024.

We compared antimicrobial resistance in *S*. Agona isolates carrying IncHI2–IncHI2A-associated ARGs (*n* = 149) with those lacking these resistance determinants (*n* = 211). Isolates harboring these ARGs exhibited significantly higher resistance rates to key antimicrobials, including ampicillin (93.3%), cefotaxime (80.5%), ceftazidime (80.5%), ciprofloxacin (65.1%), gentamicin (74.5%), chloramphenicol (89.9%), trimethoprim (94.6%), sulfamethoxazole (96.6%), tetracycline (93.3%), and tigecycline (72.5%) (Table S4). Notably, 98.0% of these isolates were classified as MDR, whereas only 0.5% of those without IncHI2–IncHI2A-associated ARGs met the MDR definition. These findings highlight the critical role of IncHI2–IncHI2A plasmids and the RGIs in the dissemination of MDR phenotypes in *S*. Agona. Although no known ARGs for azithromycin, nalidixic acid, or tigecycline resistance were identified, MDR isolates displayed substantial resistance to these antimicrobials, likely due to *ramAp* (6).

Our study highlights the notable emergence and clonal expansion of MDR *S*. Agona strains, particularly those harboring chromosomally integrated resistance genomic islands, with RGI_SA11 being especially prominent. The integration of ARGs from IncHI2–IncHI2A plasmids into the chromosome may facilitate the persistence and dissemination of resistance traits within *S*. Agona populations. The observed genetic diversity among the isolates suggests that horizontal gene transfer and clonal expansion contribute to the spread of MDR *S*. Agona. Moreover, the widespread distribution of similar IncHI2–IncHI2A plasmids across various *Salmonella* serovars and *Enterobacteriaceae* species highlights the potential for broad dissemination of resistance genes across bacterial populations. These findings emphasize the urgent need for enhanced surveillance, strict antimicrobial stewardship, and effective control strategies to prevent further spread of MDR *Salmonella* strains.

## Data Availability

The whole genome sequences of 72 ESC-resistant *S*. Agona isolates analyzed in this study have been deposited in the National Center for Biotechnology Information under BioProject accession number PRJNA478278. The specific accession numbers for each isolate are provided in Table S3.

## SUPPLEMENTAL MATERIAL

Supplemental figures spectrum.02979-24-s0001.pdf Fig. S1 to S3.

Supplemental tables spectrum.02979-24-s0002.xlsx Tables S1 to S4.

ASM does not own the copyrights to Supplemental Material that may be linked to, or accessed through, an article. The authors have granted ASM a non-exclusive, world-wide license to publish the Supplemental Material files. Please contact the corresponding author directly for reuse.
