# Supplementary material for: Clonal expansion of chromosome-borne CTX-M-55 extended-spectrum β-lactamase-producing Salmonella enterica serovar Agona, Taiwan
Source: Microbiol Spectr. 2025 Mar 19;13(5):e02979-24. doi: 10.1128/spectrum.02979-24 (PMC12054066; doi:10.1128/spectrum.02979-24)
Supplement: Supplemental figures — Fig. S1 to S3. [file spectrum.02979-24-s0001.pdf]

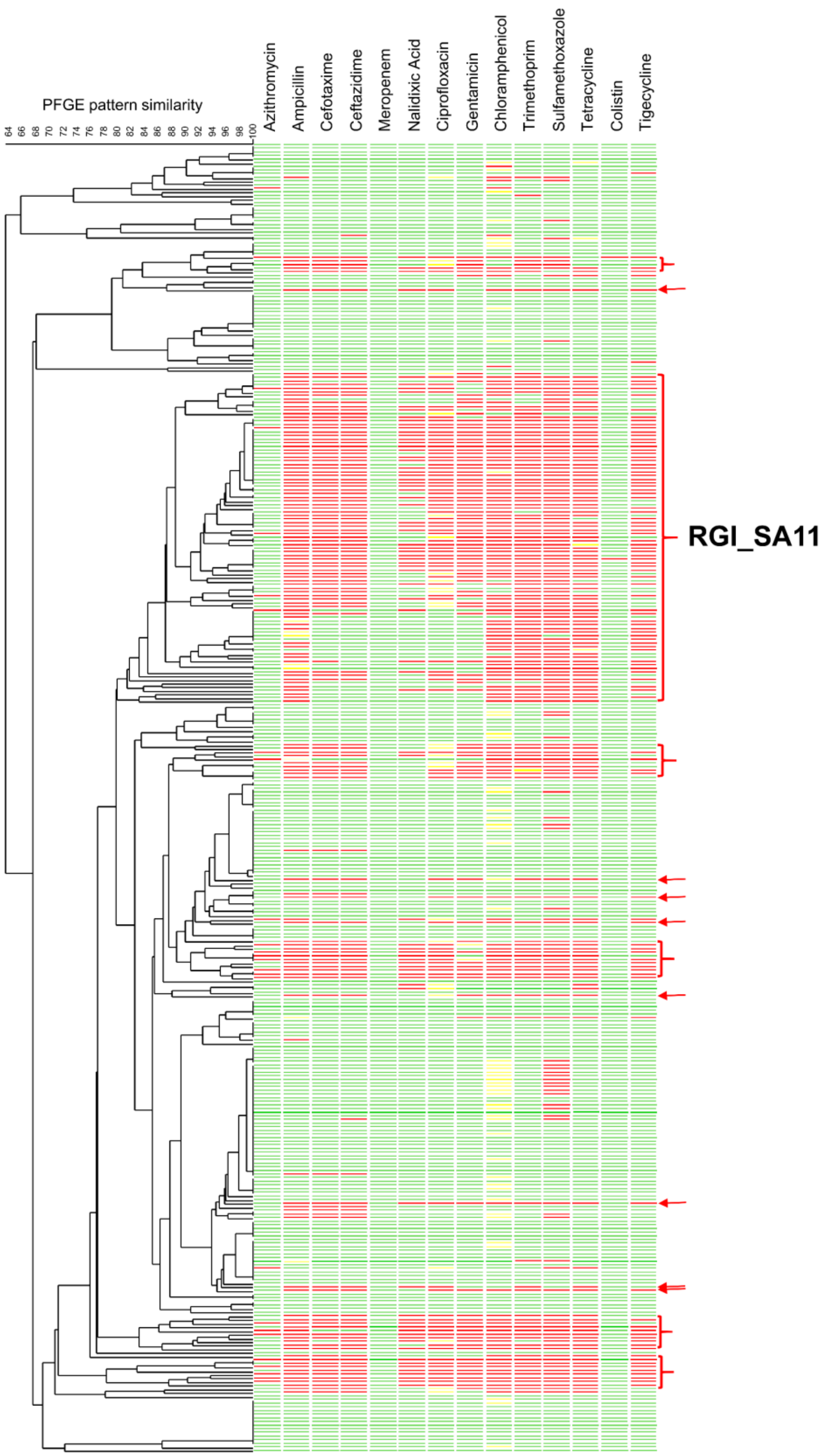
PFGE pattern similarity
64
66
68
70
72
74
76
78
80
82
84
86
88
90
92
94
96
98
100
Azithromycin
Ampicillin
Cefotaxime
Ceftazidime
Meropenem
Nalidixic Acid
Ciprofloxacin
Gentamicin
Chloramphenicol
Trimethoprim
Sulfamethoxazole
Tetracycline
Colistin
Tigecycline
RGI_SA11

Figure S1. Phylogenetic tree constructed with pulsed-field gel electrophoresis (PFGE) patterns of *S.* Agona isolates collected between 2021 and 2024. The horizontal distances represent the degree of genetic similarity, with shorter distances indicating closer relatedness between isolates. Antimicrobial resistance profiles are indicated by red lines for resistance, yellow lines for intermediate resistance, and green lines for susceptibility. ESC-resistant, multidrug-resistant isolates are marked with red arrows or red brackets. The RGI_SA11 cluster comprises both ESC-resistant and non-ESC-resistant isolates, with the latter losing the bla_{CTX-55} gene due to DNA deletion within RGI_SA11.

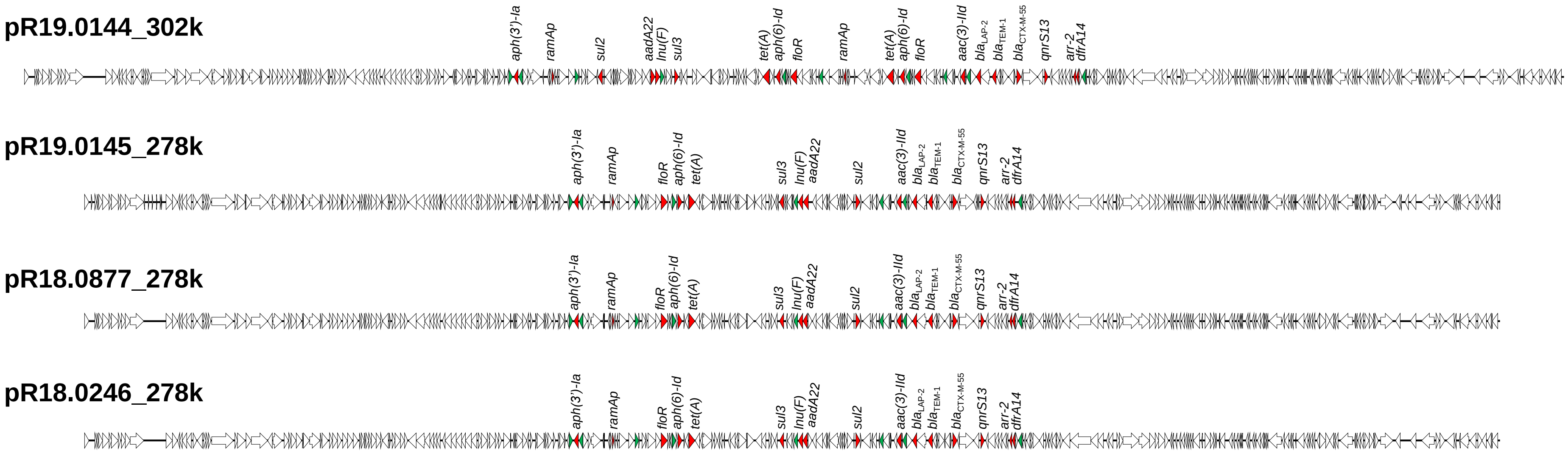

Figure S2. Genetic maps of four IncHI2-IncHI2A plasmids from *Salmonella enterica* isolates: pR19.0144_302k (from *S.* Agona R19.0144), pR19.0145_278k (*S.* Goldcoast R19.0145), pR18.0877_278k (*S.* Goldcoast R18.0877), and pR18.0246_278k (*S.* Agona R18.0246). Isolates R19.0144 and R19.0145 were collected from the same patient. Open reading frames (ORFs) corresponding to resistance genes and *ramAp* are marked in red, while ORFs linked to insertion sequences IS26 or its variant IS15D1 are highlighted in green.

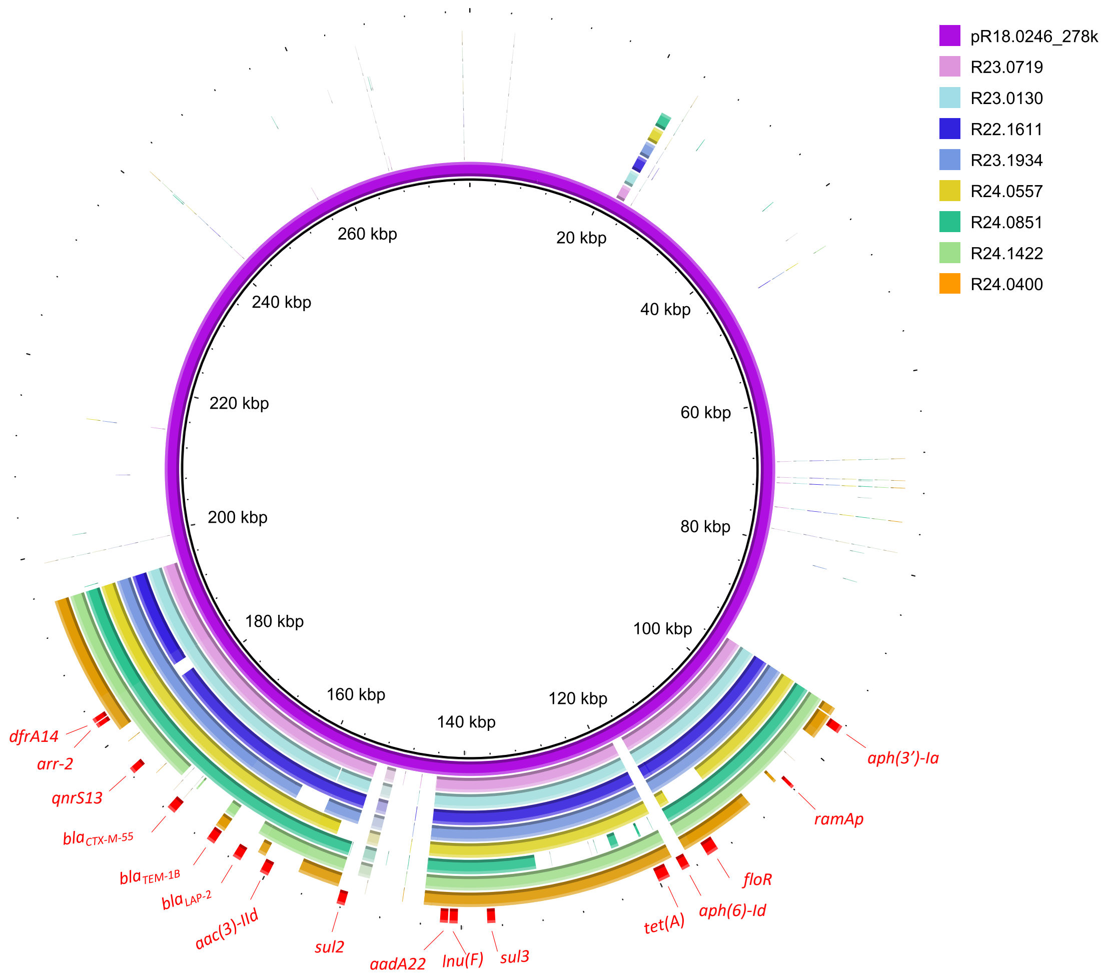

Figure S3. Genetic maps of eight variants of *S.* Agona resistance genomic island 11 (RGI_SA11) from the indicated isolates. Plasmid pR18.0246_278k was used as the reference for comparative analysis.
